# The lymph node ratio predicts cancer-specific survival of node-positive non-small cell lung cancer patients: a population-based SEER analysis

**DOI:** 10.1186/s13019-020-01390-x

**Published:** 2021-01-19

**Authors:** Liu Kai, Chen Zhoumiao, Xu Shaohua, Chen Zhao, Li Zhijun, He Zhengfu, Cai Xiujun

**Affiliations:** 1 Department of Thoracic Surgery, Sir Run Run Shaw Hospital, School of Medicine, Zhejiang University, Hangzhou, 310016 Zhejiang Province China; 2 Department of surgery, Sir Run Run Shaw Hospital, School of Medicine, Zhejiang University, 3 east qing chun road, Hangzhou, 310016 Zhejiang Province China

**Keywords:** Node-positive, Non-small cell lung cancer, Lymph node ratio, Survival

## Abstract

**Background:**

Lymph node ratio (LNR) has been suggested to be an effective prognostic tool for stratifying non-small cell lung cancer (NSCLC) cases. In this study, we sought to determine cancer-specific survival (CCS) of NSCLC cases from the SEER registry and used the X-tile method to optimize CCS-based LNR cut-off points for prognostic stratification of node-positive NSCLC.

**Methods:**

CSS and other clinicopathologic variables were retrieved from the SEER registry. Kaplan-Meier methods were used to calculate CSS. The optimal cut-off points for LNR classification were determined by the X-tile approach. Multivariate Cox regression analysis was performed to identify independent risks of CSS.

**Results:**

Totally 11,341 lung cancer patients were included. Their median CSS was 22 months (range 0,143). The median LNR was 0.22 (Q1,Q3: 0.11, 0.50). X-tile analysis showed that the optimal LNR cut-off points were 0.28 and 0.81, dividing the cohort into low (LNR1 ≤ 0.28; *n* = 6580, 58%), middle (0.28 < LNR2 < 0.81; *n* = 3025, 26.7%), and high (LNR3 > 0.81; *n* = 1736, 15.3%) subsets. Kaplan-Meier analysis showed that patients with a low LNR had a significantly higher CCS versus patients with middle or high LNR (*P* < 0.001). Multivariate competing risks regression analysis revealed that LNR was an independent and significant adverse predictor of CSS (LNR2 vs. LNR1: SHR: 1.56, 95%CI: 1.47,1.67, *P* < 0.001; LNR3 vs. LNR1: SHR: 2.54, 95%CI: 2.30,2.80, *P* < 0.001).

**Conclusions:**

LNR is an independent prognostic factor of node-positive NSCLC and its optimal cut-off values established using the robust x-tile method effectively define subpopulations of node-positive NSCLC cases, which is important in guiding selection of treatment strategies clinically.

**Supplementary Information:**

The online version contains supplementary material available at 10.1186/s13019-020-01390-x.

## Background

Lung cancer currently remains the most frequently diagnosed cancer, accounting for 11.6% of all cancer cases and is the leading cause of cancer death, contributing to 18.4% of total cancer deaths globally [1]. Non-small cell lung cancer (NSCLC) is the most common type of lung cancer, and is traditionally managed by surgical resection, radiotherapy, and chemotherapy. For patients with primary non-metastatic NSCLC, accurate staging of lymph node status is important for both prognosis and treatment decisions on the appropriateness and timing of surgery, radiotherapy and systemic therapy [2].

The classical tumor, lymph node, and metastasis (TNM) staging system remains the most convenient, reliable and acknowledged predictor of outcome of NSCLC patients. However, positive node category (pN), which is based on the number of involved lymph nodes, is affected by adequacy of lymph nodes retrieved or examined. In addition, pN is affected by age, tumor location, T stage, extent of lymphadenectomy and diligence of the pathologist [3]. Though the number of lymph nodes examined has been correlated with the survival of NSCLC patients [4], and despite improvement in lymph node assessment over the decade, the number of lymph nodes examined still varies widely by institutions [5] or across regions in the USA [4].

Adjuvant chemotherapy is routinely recommended for NSCLC patients with signs of lymph node metastasis [6] and has shown clinically demonstrable survival benefits [7]. In node-positive NSCLC cases, the lymph node ratio (LNR), defined as the ratio of positive lymph nodes to total retrieved or examined lymph nodes, has been suggested to be a more effective prognostic stratification tool [4]. Though efforts have been made to determine the prognostic significance of LNR classification in NSCLC cases, these studies are limited by inadequate study population size and lack of robustness in the methods to identify the cut-off values of LNR [8–15].

To resolve these issues, in this study, we sought to determine cancer-specific survival (CCS) of 11,341 NSCLC cases from the North American Surveillance Epidemiology and End Results (SEER) database and used the X-tile method to optimize CCS-based cut-off points of LNR. We further used the optimized LNR cut-off values for prognostic stratification of node-positive NSCLC cases.

## Methods

### Data source and patients

The SEER database abstracts data from 18 geographically diverse populations covering approximately 28% of the USA population. Data on lung cancer cases between 2004 and 2015 were retrieved using the SEER*Stat software Version 8.3.2 (http://seer.cancer.gov/seerstat). We selected treatment-naïve adult lung cancer patients (≥18 years of age) who underwent surgical resection of the primary tumor with at least one histologically retrieved lymph node using the International Classification of Diseases for Oncology, third edition diagnosis criteria (ICD-O-3). We retrieved data of patients with pathologically proven lung adenocarcinoma (ICD-O-3 codes 8140, 8141, 8147, 8250–8255, 8260, 8310, 8323, 8480, 8481, 8490, 8570–8574), lung squamous cell carcinoma (ICD-O-3 codes 8052, 8070–8076, 8078, 8083), large cell carcinoma of the lungs (ICD-O-3 codes 8012–8014), lung adenosquamous carcinoma (ICD-O-3 code 8560), and non-small cell carcinoma (ICD-O-3 code 8046) of the lungs and bronchus (ICD-O-3 C34.0–34.9). We excluded patients whose primary tumor was not located in the lungs and patients with incomplete clinicopathologic data. Patient data was reviewed and staged according to the American Joint Committee on Cancer (AJCC) Classification, 7th Edition.

This study was approved by the ethics committee of Sir Run Run Shaw Hospital, School of Medicine, Zhejiang University, Hangzhou, Zhejiang, China and patient consent was not required because of the nature of the study.

### Data retrieval

We retrieved the following variables from the SEER database: age at diagnosis, gender, race, primary site of the tumor, histological subtype, grade, pT and pN classification, the number of retrieved lymph nodes for histological examination of lymph node metastasis status, the number of positive lymph nodes, time, and CSS. Cause-specific death was death due to lung cancer. The primary outcome of the study was LNR-stratified CSS and the secondary outcome was LNR-stratified CSS of node-positive NSCLC cases.

### Statistical analysis

For descriptive statistics, absolute number with proportion for categorical variables, mean and standard deviation (SD) for continuous variables with Gaussian distribution and median and the 25th percentile (Q1) and 75th percentile (Q3) for continuous variables with non-normal distribution were used, respectively. The optimal cut-off points for LNR classification were determined by the X-tile approach [16] and proper LNR cut-off values were then used for a novel LNR subclassification scheme. Chi-square test was used to compare LNR distribution by histopathological parameters (including pT, pN, and the number of retrieved/positive/negative lymph nodes).

Kaplan-Meier methods were used to calculate CSS and the log-rank test was used for statistical comparisons. Competing risks regression analysis was used according to the model of Fine and Gray to calculate the cumulative probability of lung cancer-specific mortality using death from non-lung cancer as the competing variable. Multivariate Cox regression analysis incorporating LNR classification or pN as a covariate was carried out to identify independent risk factors of CSS. Meanwhile, Akaike information criteria (AIC) was calculated to evaluate fitness between the model incorporating LNR classification versus the model incorporating pN category with a lower value of AIC indicating the better fit of a statistical model. Finally, multivariate competing risks regression analysis incorporating LNR classification versus the model incorporating pN category as a covariate was performed to examine the association between CSS and independent prognostic factors. Hazard ratios (HRs) or sub-distribution hazard ratios (SHRs) were calculated with 95% confidence intervals (CIs). Statistical analyses were performed using the statistical package R, Version 3.1.0 (R Project for Statistical Computing, Vienna, Austria) with a *P* value < 0.05 considered statistically significant.

## Results

### Demographic and baseline characteristics of the study population

**Fig. 1:**
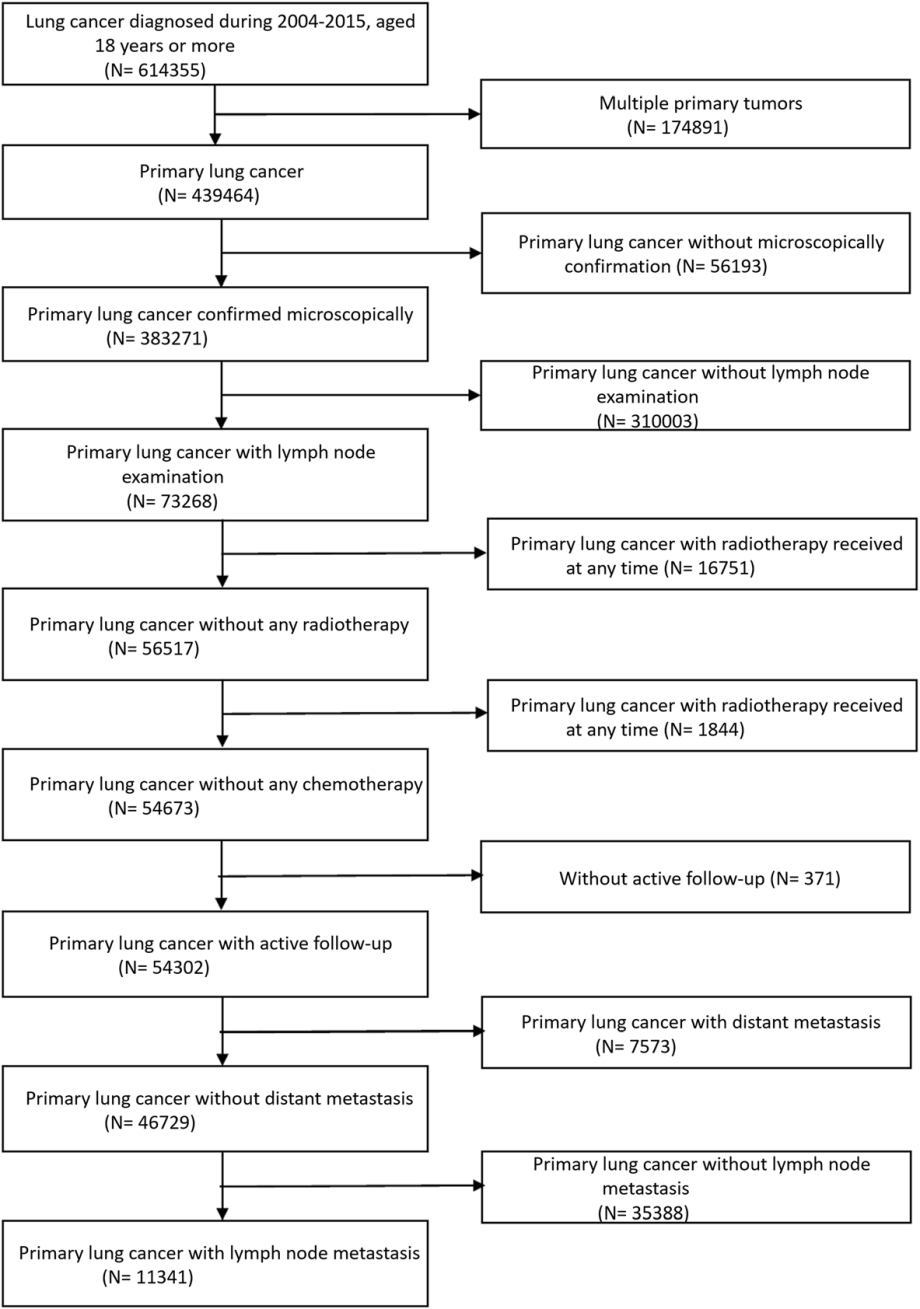
The study flowchart

The study flowchart is shown in Fig. 1. Totally 614,355 patients were diagnosed with lung cancer during the study period; 11,341 of them met the eligibility criteria and were included in the current study. Slightly more than half (53.1%) of them were male. The upper lobes of the lungs were the most frequent primary site of NSCLC (53.4%); adenocarcinoma (54.3%) was the most common histological subtype of NSCLC. Furthermore, 56.3, 38.5 and 5.3% of NSCLC cases had N1, N2 and N3 disease, respectively. The demographic and baseline characteristics of the study population are shown in Table 1.

**Table 1:** Demographic and baseline characteristics of NSCLC cases from the Surveillance, Epidemiology, and End Results (SEER) database between 2004 and 2015

Characteristics	No. (%)
Male gender	6016 (53.1)
Race	
White	9335 (82.3)
Black	1087 (9.6)
Others	919 (8.1)
Age, years	
> 60	8138 (71.8)
Primary site	
Upper lobe	6054 (53.4)
Lower lobe	3757 (33.1)
Main bronchus	255 (2.3)
Middle lobe	532 (4.7)
Overlapping/NOS	743 (6.6)
Grade	
I/II	4643 (48.0)
III/IV	5033 (52.0)
Histology	
Adenocarcinoma	6159 (54.3)
Squamous	3134 (27.6)
Others	2048 (18.1)
T stage	
T1	2842 (25.9)
T2	5892 (53.6)
T3	665 (6.1)
T4	1596 (14.5)
N stage	
N_N1	6384 (56.3)
N_N2	4362 (38.5)
N_N3	595 (5.3)

### CSS

**Fig. 2:**
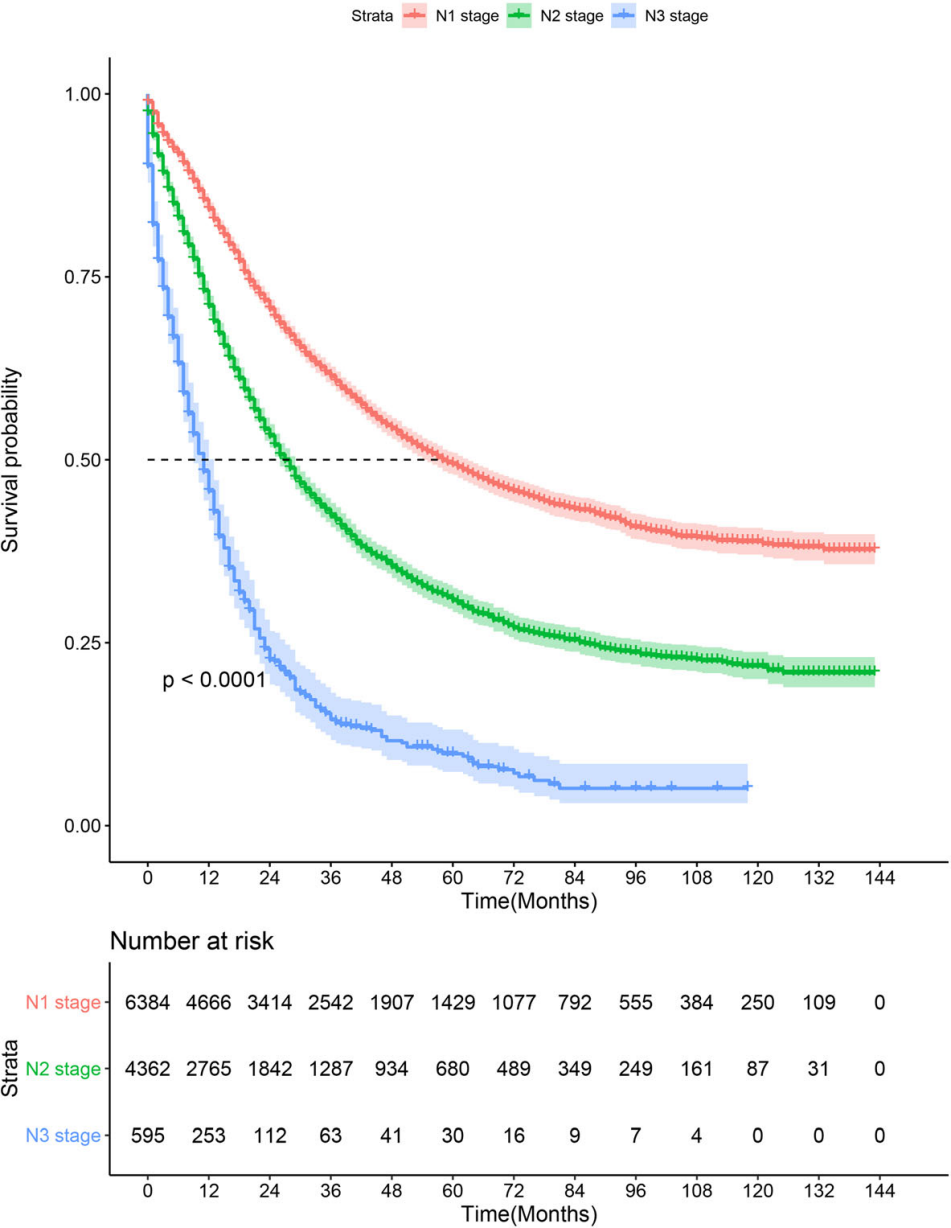
Cancer-specific survival stratified by pN of patients with lung cancer in the Surveillance, Epidemiology, and End Results database

The patients were followed up for median duration of 22 months (Q1,Q3: 8, 48). Totally 5757 patients died due to NSCLC during the follow up. The median CSS of the study population was 22 months (range 0,143). The median CSS of N1, N2 and N3 NSCLC cases was 26 months (range 0, 143), 19 months (range 0, 143), and 9 months (range 0, 143), respectively (Fig. 2).

### LNR and N stage

**Fig. 3:**
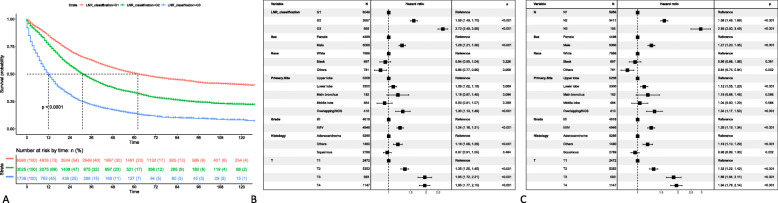
Cancer-specific survival (**a**) of patients with lung cancer in the SEER database are stratified using the optimal cut-off points (0.28 and 0.81) of lymph node ratio (LNR) by X-tile analysis: LNR1 ≤ 0.28; 0.28 < LNR2 < 0.81; LNR3 > 0.81. Forest plots showing results of multivariate Cox regression analysis using LNR as a covariate for prediction of CSS (**b**). Forest plots showing results of multivariate Cox regression analysis using pN as a covariate for prediction of CSS (**c**). On the basis of the Cox model, prespecified subgroup analyses of covariates of interest were conducted to estimate hazard ratios (HR) with 95% confidence intervals (CI) and to test for interaction among subgroups with the use of two-sided *P* values. A HR of less than 1 implies a lower risk of death

The median number of retrieved lymph nodes were 9 (Q1,Q3: 4,15) and the median number of positive retrieved lymph nodes were 2 (Q1,Q3: 1,3). The median LNR was 0.22 (Q1,Q3: 0.11, 0.50). X-tile analysis of CSS data of NSCLC cases from the SEER registry showed that the optimal LNR cut-off points were 0.28 and 0.81, which divided the entire cohort into low (LNR1 ≤ 0.28; *n* = 6580, 58%), middle (0.28 < LNR2 < 0.81; *n* = 3025, 26.7%), and high (LNR3 > 0.81; *n* = 1736, 15.3%) subsets. Kaplan-Meier analysis further showed that patients with a low LNR had a significantly higher CCS versus patients with middle or high LNR (*P* < 0.001) (Fig. 3a). Chi-square test showed a significant association of LNR with the median number of retrieved lymph nodes as well as the median number of positive and negative lymph nodes (*P* < 0.001) and pT and pN stage (Chi-square test, *P* < 0.001) (Supplementary Table 1). Our multivariate Cox regression analysis using LNR as a covariate further showed that higher LNR was an independent and significant adverse predictor of CSS (LNR3 vs. LNR1: HR: 2.73 95%CI 2.49, 2.99; *P* < 0.001) (Fig. 3b).

Our multivariable competing risks regression analysis, with inclusion of LNR and N stage, respectively, revealed that LNR was an independent and significant adverse predictor of CSS (LNR2 vs. LNR1: SHR: 1.56, 95%CI: 1.47,1.67, *P* < 0.001; LNR3 vs. LNR1: SHR: 2.54, 95%CI: 2.30,2.80, *P* < 0.001) (Table 2). Moreover, our multivariate Cox regression analysis using pN as a covariate showed that higher pN was an independent and significant adverse predictor of CSS (N2 vs. N1: HR: 1.58, 95%CI 1.49, 1.68; *P* < 0.001 and N3 vs. N1: HR: 2.95, 95%CI 2.50, 3.49; *P* < 0.001) (Fig. 3c). Furthermore, our multivariable competing risks regression analysis, with inclusion of LNR and N stage, respectively, revealed that pN was an independent and significant adverse predictor of CSS (N2 vs. N1: SHR: 1.54, 95%CI: 1.45,1.63, *P* < 0.001; N3 vs. N1: SHR: 2.81, 95%CI: 2.32,3.41,*P* < 0.001) (Table 2). Assessment of the two multivariate Cox models incorporating LNR and pN as covariates showed that LNR had a lower AIC value (LNR vs pN: 2137.4 vs 2391.7 for OS and 2064.3 vs 2301.3 for CSS) and was, hence, a better prognostic indicator of CSS and OS than pN.

**Table 2:** Competing risks regression analysis with separate inclusion of the lymph node ratio (LNR) and pN stage as a covariate

Characteristics	SHR	*P*	SHR	*P*
LNR classification				
LNR1	1(Reference)			
LNR2	1.56 (1.47–1.67)	< 0.001		
LNR3	2.54 (2.30–2.80)	< 0.001		
N stage				
N1			1 (Reference)	
N2			1.54 (1.45–1.63)	< 0.001
N3			2.81 (2.32–3.41)	< 0.001
Sex				
Female	1(Reference)		1(Reference)	
Male	1.23 (1.16–1.30)	< 0.001	1.21 (1.134–1.29)	< 0.001
Race				
White	1(Reference)		1(Reference)	
Black	0.94 (0.85–1.05)	0.262	0.97 (0.87–1.07)	0.506
Others	0.90 (0.81–1.00)	0.040	0.88 (0.79–0.98)	0.020
Primary site				
Upper lobe	1(Reference)		1(Reference)	
Lowerlobe	1.08 (1.01–1.15)	0.018	1.11 (1.04–1.18)	0.001
Mainbronchus	1.20 (0.96–1.49)	0.103	1.21 (0.98–1.51)	0.083
Middlelobe	0.93 (0.81–1.06)	0.290	1.03 (0.91–1.18)	0.625
Overlapping/NOS	1.28 (1.10–1.49)	0.001	1.30 (1.12–1.52)	< 0.001
Grade				
I/II	1(Reference)		1(Reference)	
III/IV	1.23 (1.16–1.31)	< 0.001	1.25 (1.18–1.33)	< 0.001
Histology				
Adenocarcinoma	1(Reference)		1(Reference)	
Squamous	0.92 (0.86–0.99)		0.91 (0.84–0.97)	0.008
Others	1.15 (1.05–1.25)	0.002	1.14 (1.04–1.24)	0.003
T stage		0.030		
T1	1(Reference)			
T2	1.32 (1.23–1.412)	< 0.001	1.29 (1.199–1.38)	< 0.001
T3	1.85 (1.62–2.12)	< 0.001	1.78 (1.56–2.04)	< 0.001
T4	1.82 (1.64–2.01)	< 0.001	1.82 (1.64–2.01)	< 0.001

*LNR* Lymph node ratio, *SHR* sub-distribution hazards ratio

### Prognostic stratification of pN diseases by LNR

We further sought to elucidate the relationship between LNR and pN stage as lymph node involvement is one of the most critical determinants of clinical outcomes and dictates treatment strategy of resectable NSCLC patients. A significantly greater proportion of patients whose LNR was ≤0.28 had N1 disease (LNR1 69.8% vs. LNR3:18.0%; *P* < 0.001). Meanwhile, a significantly greater proportion of patients whose LNR was > 0.81 had N3 stage (LNR3: 26.8% vs. LNR1: 1.0% and LNR2: 2.1%; *P* < 0.001).

**Fig. 4:**
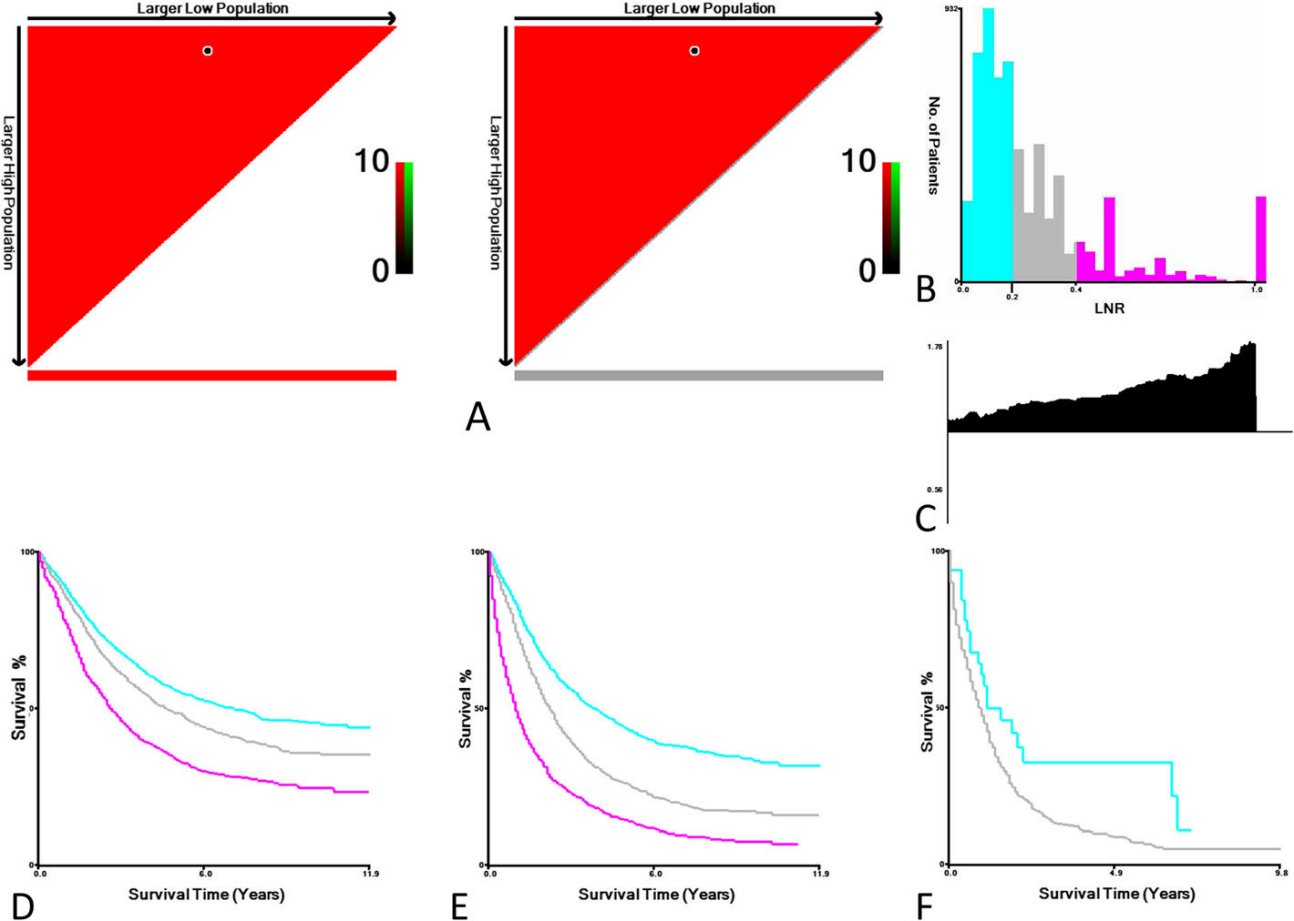
X-tile analysis of cause-specific survival (CSS) data of node positive (N1) NSCLC cases from the SEER registry. **a** The plot shows the [2] log-rank values produced when dividing the cohort with two cut-points,0.17 and 0.38, producing high, middle, and low subsets (low subset: blue, middle subset: gray, high subset: magenta). The *X*-axis represents all potential cut-points from low to high (*left to right*) that define a low subset, whereas the *Y*-axis represents cut-points from high to low (*top to bottom*), that define a high subset. The *arrows* represent the direction in which the low subset (*X*-axis) and the high subset (*Y*-axis) increase in size. *Red coloration* of cut-points indicates an inverse correlation with CSS, whereas *green coloration* represents direct associations. The optimal cut-point occurs at the *brightest pixel* (*green* or *red*). The cut-point in (**a**) is shown on a histogram of the entire cohort (**b**), the relative risk (RR) is displayed in (**c**) and a Kaplan-Meier plot (**d**) is drawn. *P < 0.001. A Kaplan-Meier plot is drawn for the CSS of the N2 (**e**) and N3 (**f**) subpopulation stratified by the LNR. *P < 0.001

We then analyzed by X-tile the CSS data of N1 NSCLC cases and found that the optimal LNR cut-off points for the N1 NSCLC subpopulation were 0.17 and 0.38. The N1 subpopulation was divided by the two cut-off points into low (LNR1 ≤ 0.17; *n* = 3425, 53.7%), middle (0.17 < LNR2 < 0.38; *n* = 1832, 28.7%), and high (LNR3 > 0.38; *n* = 1127, 17.7%) subsets (Fig. 4a). X-tile analysis of CCS data of N1 stage NSCLC cases from the SEER registry revealed a continuous distribution based on LNR (Fig. 4a and b) and higher LNR was associated with higher RR (Fig. 4c). Kaplan-Meier analysis further showed that patients with a low LNR had a significantly higher CCS (log rank test, *P* < 0.001) (Fig. 4d).

X-tile analysis of the CSS data of N2 NSCLC cases revealed that the optimal cut-off points of LNR for the N2 subpopulation were 0.30 and 0.80. The two cut-off points divided the N2 subpopulation into low (LNR1 ≤ 0.28; *n* = 1921, 44.0%), middle (0.28 < LNR2 < 0.83; *n* = 1504, 34.5%), and high (LNR3 > 0.83; *n* = 937, 21.5%) subsets (Supplementary Figure 1A to C). Kaplan-Meier analysis showed that patients with a low LNR had a significantly higher CCS versus patients with middle or high LNR (log rank test, *P* < 0.001) (Fig. 4e). Furthermore, X-tile analysis of the CSS data of N3 NSCLC cases showed that the optimal cut-off point of LNR for the N3 subpopulation was 0.03. The N3 subpopulation was categorized by the cut-off point into low (*n* = 33, 5.6%) and high (*n* = 562, 94.5%) subsets (Supplementary Figure 2A to C). Kaplan-Meier analysis further revealed that patients with a low LNR had a significantly higher CCS versus patients with high LNR (log rank test, *P* < 0.001) (Fig. 4f).

## Discussion

In this study, we carried out a population-based analysis of 11,341 NSCLC cases in the SEER registry. Our analysis has shown that the LNR is a powerful independent prognostic predictor of CSS of NSCLC patients. Importantly, our study has demonstrated that the LNR is a significant and independent predictor of CSS of node positive NSCLC patients, which could aid risk stratification and guide treatment strategy of resectable node positive NSCLC cases.

However, pN category, which is based on the number of involved lymph nodes, is affected by adequacy of lymph nodes retrieved or examined. In addition, pN is affected by age, tumor location, T stage, extent of lymphadenectomy and diligence of the pathologist [3]. Though the number of lymph nodes examined has been correlated with the survival of NSCLC patients [4], and despite improvement in lymph node assessment over the decade, the number of lymph nodes examined still varies widely by institutions [5] or across regions in the USA [4].

The classical TNM staging system provides a convenient and reliable indicator of outcome of NSCLC patients; however, it has become increasingly defined that NSCLC patients may have different prognoses even with the same T, N and M stages, especially those in the N2 group [17].The present pN classification is based on the idea that lymph node metastasis initially occurs in those neighboring the primary tumor, and then sequentially spreads to more distant lymph nodes such as the mediastinal lymph nodes. However, metastasis may initially occur in the mediastinal lymph nodes without N1 metastasis in about one-quarter of patients [18–21], and skip N2 patients had a better prognosis than non-skip N2 patients [22].

The IASLC has proposed a four-category classification of lymph node status: N0, single zone N1, multiple zones N1/single zone N2 and multiple zones N2, and suggested that the overall disease burden, rather than just the anatomical location of lymph node involvement, may influence the outcome [2]. Although substantial efforts have been made to validate the new four-category classification, unfortunately, the International Staging Committee prudently decided to maintain the current N descriptors in the latest eighth edition until future analyses of larger and more homogeneous databases become available. The presence of metastatic lymph nodes is one of the most important determinants of prognosis of NSCLC cases. However, the number of lymph nodes and number of stations examined are two surrogates for staging [23]. These two measures depend on the actions of surgeons during the operation (the collection of hilar and mediastinal lymph nodes) and pathologists after the operation (the retrieval of intrapulmonary nodes and throughout examination of all provided specimens) [24]. Consequently, the number of lymph nodes removed and examined may not be optimal for reliable prognostic stratification and varies widely by institutions [5] or across regions in the USA. For these reasons, the concept of the LNR as a prognostic factor in NSCLC has gained increasing attention. This study has demonstrated that the LNR is an independent prognostic factor of CSS and could stratify different N stage NSCLC cases. To our knowledge, this is the largest reported study assessing the value of the LNR in node-positive NSCLC cases.

Several studies have recently demonstrated the prognostic role of the LNR in NSCLC patients. Matsuguma et al. reported that LNR and the number of metastatic lymph nodes may be more effective prognostic indicators than the current pN classification based on the location of metastatic lymph nodes. Moreover, they proposed that a larger number of resected negative lymph nodes was found to be a significantly favorable prognostic factor in node-positive patients [8]. Similarly, Ludwig et al. showed that postoperative survival of NSCLC patients was associated with the number of lymph nodes resected during surgery, and they recommended that the optimal number of resected lymph nodes should be 11 to 16. In a study [4] of 38,806 NSCLC cases from the SEER registry and 5706 NSCLC cases from a Chinese registry, Liang et al. found that a greater number of examined lymph nodes is associated with more accurate node staging and better long-term survival of resected NSCLC and recommended 16 examined lymph nodes as the cut point for prognostic stratification postoperatively for patients with declared node-negative disease.

However, lymph nodes are sometimes resected as fragments, and the pN classification could be compromised by the presence of systematic inter-institutional differences in the surgical approach with regards to inspecting, sampling and resecting nodes [25].Taking this into consideration, we suggested that LNR classification is better than pN classification because, if nodes are fragmented or more nodes were retrieved, both the number of metastatic lymph nodes and the total number of lymph nodes will increase. Therefore, LNR could minimize the effects of confounding factors such as lymph node fragmentation or interindividual differences in the number of lymph nodes in the lymphatic chain. Chiappetta et al. also identified the LNR as an independent prognostic factor for overall survival(OS) and disease-free survival(DFS) in patients undergoing curative resection of NSCLC, especially for those in pathological stages II and III(TNM Classification of Malignant Tumours,8th edition) [26]. However, all of the above studies have included patients who received postoperative adjuvant therapy (radiotherapy or chemotherapy), which could influence the prognosis of patients greatly, suggesting that their data did not reflect the natural course and real survival duration of NSCLC. Meanwhile, in the present study, our NSCLC patients were treatment naïve. In this respect, our survival data and results are more objective and realistic than previous studies. In addition, different from previous studies on the prognostic role of LNR in NSCLC, we used the robust X-tile program to identify the optimum cutoff. Compared with cutoff which was aimed at matching the number of patients in each stage with current pN classification [8], and which was just based on experience [26], our cutoff value was more scientific and reliable.

The study has several limitations. Firstly, it was a retrospective exploratory study based on the SEER registry and the cut-off value of LNR need to be validated in an independent cohort. Another limitation is that detailed adjuvant therapy information is not available, which makes the evaluation or adjustment of treatment effect impossible. Now we are constructing our own database and we hope to validate the LNR methods in our future research by using our own data. Further large-scale prospective studies are still needed to be conducted to explore and validate the prognostic value of LNR in node-positive NSCLC patients and its predictive value in defining node-positive NSCLC subpopulations.

## Conclusions

In summary, the LNR classification is an independent prognostic factor of node-positive NSCLC and the optimal cut-off values of LNR established using the robust x-tile method effectively define subpopulations of node-positive NSCLC cases, which is important in guiding selection of treatment strategies clinically.

## Supplementary Information

**Additional file 1.**

## Data Availability

The datasets generated and/or analysed during the current study are available in the SEER repository, https://seer.cancer.gov/

## Abbreviations

AIC: Akaike information criteria
AJCC: American Joint Committee on Cancer
CCS: Cancer-specific survival
Cis: Confidence intervals
DFS: Disease-free survival
HRs: Hazard ratios
LNR: Lymph node ratio
NSCLC: Non-small cell lung cancer
OS: Overall survival
pN: positive node category
SEER: Surveillance Epidemiology and End Resul
SD: Standard deviation
SHRs: Sub-distribution hazard ratios
TNM: Tumor, lymph node, and metastasis
