# Supplementary material for: The lymph node ratio predicts cancer-specific survival of node-positive non-small cell lung cancer patients: a population-based SEER analysis
Source: J Cardiothorac Surg. 2021 Jan 19;16:13. doi: 10.1186/s13019-020-01390-x (PMC7814600; doi:10.1186/s13019-020-01390-x)
Supplement: Supplementary file 1 — Additional file 1. [file 13019_2020_1390_MOESM1_ESM.docx]

**Supplementary** **methods**

Kaplan-Meier methods were used to calculate OS and the log-rank test was used for statistical comparisons. Competing risks regression analysis was used according to the model of Fine and Gray to calculate the cumulative probability of lung cancer-specific mortality using death from non-lung cancer as the competing variable. Multivariate Cox regression analysis incorporating LNR classification or pN as a covariate was carried out to identify independent risk factors of OS. Meanwhile, Akaike information criteria (AIC) was calculated to evaluate fitness between the model incorporating LNR classification *versus* the model incorporating pN category with a lower value of AIC indicating the better fit of a statistical model. Finally, multivariate competing risks regression analysis incorporating LNR classification *versus* the model incorporating pN category as a covariate was performed to examine the association between OS and independent prognostic factors. Hazard ratios (HRs) or sub-distribution hazard ratios (SHRs) were calculated with 95% confidence intervals (CIs). Statistical analyses were performed using the statistical package R, Version 3.1.0 (R Project for Statistical Computing, Vienna, Austria) with a *P* value <0.05 considered statistically significant.

**Supplementary data**

**OS**

The median OS stratified by N stage is shown in **Supplementary Figure 3.** Patients with a low LNR had a significantly higher OS *versus* patients with middle or high LNR (log rank test, *P*< 0.001) (**Supplementary Figure 4A**). Our multivariate Cox regression analysis using LNR as a covariate showed that higher LNR was also an independent and significant adverse predictor of OS (LNR3 *vs.* LNR1: HR: 2.47 95%CI 2.27, 2.69; *P* < 0.001) (**Supplementary Figure 4B**).

**LNR and N stage**

X-tile analysis of OS data of N1 stage NSCLC cases showed that the optimal cut-off points of LNR for the N1 NSCLC subpopulation were 0.10 and 0.40 (**Supplementary Figure 5A and B**). Kaplan-Meier analysis further showed that patients with a low LNR had a significantly higher OS *versus* patients with middle or high LNR (log rank test, *P*< 0.001) (**Supplementary Figure 5C**). Furthermore, the results of X-tile analysis of OS data of N2 and N3 stage NSCLC cases are shown in **Supplementary Figure 6** and **7**.

**Supplementary figure legends**

**Supplementary Figure 1** X-tile analysis of cause-specific survival (CSS) data of node positive (N2) NSCLC cases from the SEER registry. (A) The plot shows the χ^2^ log-rank values produced when dividing the cohort with two cut-points,0.30 and 0.80, producing high, middle, and low subsets (low subset: blue, middle subset: gray, high subset: magenta). The *X*-axis represents all potential cut-points from low to high (*left to right*) that define a low subset, whereas the *Y*-axis represents cut-points from high to low (*top to bottom*), that define a high subset. The *arrows* represent the direction in which the low subs t (*X*-axis) and the high subset (*Y*-axis) increase in size. *Red coloration* of cut-points indicates an inverse correlation with CSS, whereas *green coloration* represents direct associations. The optimal cut-point occurs at the *brightest pixel* (*green* or *red*). The cut-point in (A) is shown on a histogram of the entire cohort (B), and the relative risk (RR) is displayed in (C).

**Supplementary Figure 2** X-tile analysis of cause-specific survival (CSS) data of node positive (N3) NSCLC cases from the SEER registry. (A) The plot shows the χ^2^ log-rank values produced when dividing the cohort with the cut-point, 0.03, producing low and high subsets (low subset: blue, and high subset: magenta). The *X*-axis represents all potential cut-points from low to high (*left to right*) that define a low subset, whereas the *Y*-axis represents cut-points from high to low (*top to bottom*), that define a high subset. The *arrows* represent the direction in which the low subset (*X*-axis) and the high subset (*Y*-axis) increase in size. *Red coloration* of cut-points indicates an inverse correlation with CSS, whereas *green coloration* represents direct associations. The optimal cut-point occurs at the *brightest pixel* (*green* or *red*). The cut-point in (A) is shown on a histogram of the entire cohort (B), and the relative risk (RR) is displayed in (C).

**Supplementary Figure 3** Overall survival stratified by pN of patients with lung cancer in the Surveillance, Epidemiology, and End Results database.

**Supplementary Figure 4** Overall survival (A) of patients with lung cancer in the SEER database are stratified using the optimal cut-off points (0.28 and 0.81) of lymph node ratio (LNR) by X-tile analysis: LNR1 ≤ 0.28; 0.28 < LNR2< 0.81; LNR3> 0.81. Forest plots showing results of multivariate Cox regression analysis using the LNR as a covariate for prediction of OS (B). On the basis of the Cox model, prespecified subgroup analyses of covariates of interest were conducted to estimate hazard ratios (HR) with 95% confidence intervals (CI) and to test for interaction among subgroups with the use of two-sided *P* values. A HR of less than 1 implies a lower risk of death.

**Supplementary Figure 5** X-tile analysis of overall survival (OS) data of node positive N1 NSCLC cases from the SEER registry. (A) The plot shows the χ^2^ log-rank values produced when dividing the cohort with two cut-points,0.10 and 0.40, producing high, middle, and low subsets(low subset: blue, middle subset: gray, high subset: magenta). The *X*-axis represents all potential cut-points from low to high (*left to right*) that define a low subset, whereas the *Y*-axis represents cut-points from high to low (*top to bottom*), that define a high subset. The *arrows* represent the direction in which the low subset (*X*-axis) and the high subset (*Y*-axis) increase in size. *Red coloration* of cut-points indicates an inverse correlation with OS, whereas *green coloration* represents direct associations. The optimal cut-point occurs at the *brightest pixel* (*green* or *red*). The cut-point in (A) is shown on a histogram of the entire cohort (B), and a Kaplan-Meier plot (C) is drawn.

**Supplementary Figure 6** X-tile analysis of overall survival (OS) data of node positive N2 NSCLC cases from the SEER registry. (A) The plot shows the χ^2^ log-rank values produced when dividing the cohort with two cut-points,0.30 and 0.90, producing high, middle, and low subsets (low subset: blue, middle subset: gray, high subset: magenta). The *X*-axis represents all potential cut-points from low to high (*left to right*) that define a low subset, whereas the *Y*-axis represents cut-points from high to low (*top to bottom*), that define a high subset. The *arrows* represent the direction in which the low subset (*X*-axis) and the high subset (*Y*-axis) increase in size. *Red coloration* of cut-points indicates an inverse correlation with OS, whereas *green coloration* represents direct associations. The optimal cut-point occurs at the *brightest pixel* (*green* or *red*). The cut-point in (A) is shown on a histogram of the entire cohort (B), and a Kaplan-Meier plot (D) is drawn.

**Supplementary Figure 7** X-tile analysis of overall survival (OS) data of node positive N3 NSCLC cases from the SEER registry. (A) The plot shows the χ^2^ log-rank values produced when dividing the cohort with two cut-points, 0.10 and 0.40, producing high, and low subsets (low subset: blue, and high subset: magenta). The *X*-axis represents all potential cut-points from low to high (*left to right*) that define a low subset, whereas the *Y*-axis represents cut-points from high to low (*top to bottom*), that define a high subset. The *arrows* represent the direction in which the low subset (*X*-axis) and the high subset (*Y*-axis) increase in size. *Red coloration* of cut-points indicates an inverse correlation with OS, whereas *green coloration* represents direct associations. The optimal cut-point occurs at the *brightest pixel* (*green* or *red*). The cut-point in (A) is shown on a histogram of the entire cohort (B), and a Kaplan-Meier plot (D) is drawn.

**Supplementary Table 1.** Lymph node ratio (LNR) distribution by pN stage and retrieved lymph node status of NSCLC patients in the Surveillance, Epidemiology, and End Results (SEER) database

|  | Lymph node ratio (LNR) | | | | *P* |
| --- | --- | --- | --- | --- | --- |
|  | Total | ≤ 0.28 | 0.28- 0.81 | >0.81 |  |
| N(%) | 11341 | 6580(58.0%) | 3025(26.7) | 1736(15.3) |  |
| pT, n(%) |  |  |  |  | < 0.001 |
| T1 | 2842(25.9) | 1680(25.7) | 813(27.3) | 349(23.6) |  |
| T2 | 5892(53.6) | 3741(57.3) | 1628(54.6) | 523(35.3) |  |
| T3 | 665(6.1) | 432(6.6) | 152(5.1) | 81(5.5) |  |
| T4 | 1596(14.5) | 681(10.4) | 388(13.0) | 527(35.6) |  |
| pN, n(%) |  |  |  |  | < 0.001 |
| N1 | 6384(56.3) | 4593(69.8) | 1478(48.9) | 313(18.0) |  |
| N2 | 4362(38.5) | 1921(29.2) | 1483(49.0) | 958(55.2) |  |
| N3 | 595(5.3) | 66(1.0) | 64(2.1) | 465(26.8) |  |
| No. of retrieved lymph nodes |  |  |  |  |  |
| Median, Q1,Q3:) | 9(4,15) | 12(8,18) | 7(4,11) | 1(1,3) | < 0.001 |
| No. of positive lymph node(median, Q1,Q3:) | 2(1,3) | 1(1,2) | 3(2,5) | 1(1,3) | < 0.001 |
| No. of negative lymph node(median, Q1,Q3:) | 7(2,12) | 11(7,16) | 4(2,6) | 0(0,0) | < 0.001 |

*Chi-squared test
